# Multidisciplinary patient-centered management of brain metastases and future directions

**DOI:** 10.1093/noajnl/vdaa034

**Published:** 2020-03-16

**Authors:** Joshua D Palmer, Daniel M Trifiletti, Vinai Gondi, Michael Chan, Giuseppe Minniti, Chad G Rusthoven, Steven E Schild, Mark V Mishra, Joseph Bovi, Nicole Williams, Maryam Lustberg, Paul D Brown, Ganesh Rao, David Roberge

**Affiliations:** 1 Department of Radiation Oncology, The James Cancer Hospital and Solove Research Institute, The Ohio State University Wexner Medical Center, Columbus, Ohio, USA; 2 Department of Neurosurgery, The James Cancer Hospital and Solove Research Institute, The Ohio State University Wexner Medical Center, Columbus, Ohio, USA; 3 Departments of Radiation Oncology and Neurological Surgery, Mayo Clinic, Jacksonville, Florida, USA; 4 Department of Radiation Oncology, Northwestern University Feinberg School of Medicine, Chicago, Illinois, USA; 5 Radiation Oncology Consultants LLC, Chicago, Illinois, USA; 6 Northwestern Medicine Chicago Proton Center Warrenville, Chicago, Illinois, USA; 7 Department of Radiation Oncology, Wake Forest School of Medicine, Winston-Salem, North Carolina, USA; 8 Radiation Oncology Unit, UPMC Hillman Cancer Center, San Pietro Hospital FBF, Rome, Italy; 9 Department of Radiation Oncology, University of Colorado School of Medicine, Aurora, Colorado, USA; 10 Department of Radiation Oncology, Mayo Clinic Scottsdale, Phoenix, Arizona, USA; 11 Department of Radiation Oncology, University of Maryland School of Medicine, Baltimore, Maryland, USA; 12 Department of Radiation Oncology, Medical College of Wisconsin, Milwaukee, Wisconsin, USA; 13 Department of Medical Oncology, The James Cancer Hospital and Solove Research Institute at The Ohio State University Wexner Medical Center, Columbus, Ohio, USA; 14 Department of Radiation Oncology, Mayo Clinic, Rochester, Minnesota, USA; 15 Department of Neurosurgery, The University of Texas MD Anderson Cancer Center, Houston, Texas, USA; 16 Department of Radiation Oncology, Centre Hospitalier de l’ Université de Montreal, Montreal, Quebec, Canada

**Keywords:** brain metastasis, radiosurgery, surgery, systemic therapy, treatment overview

## Abstract

The incidence of brain metastasis is increasing as improvements in systemic therapy lead to increased survival. This provides new and challenging clinical decisions for patients who are trying to balance the risk of recurrence or progression with treatment-related side effects, and it requires appropriate management strategies from multidisciplinary teams. Improvements in prognostic assessment and systemic therapy with increasing activity in the brain allow for individualized care to better guide the use of local therapies and/or systemic therapy. Here, we review the current landscape of brain-directed therapy for the treatment of brain metastasis in the context of recent improved systemic treatment options. We also discuss emerging treatment strategies including targeted therapies for patients with actionable mutations, immunotherapy, modern whole-brain radiation therapy, radiosurgery, surgery, and clinical trials.

Brain metastases (BMs) are the most common intracranial neoplasm in adults and are 10 times more frequent than primary brain tumors.^1^ BMs occur in 20–40% of all cancer patients and are associated with a poor median survival time of 6–12 months.^2^ The most common primary tumors in patients with BM are lung, breast, melanoma, colorectal, and renal tumors.^2,3^ The frequency of BMs appears to be increasing as a result of improved neuroimaging modalities and longer survival of patients with metastatic disease due to improved systemic therapy treatment options.

BMs are distributed along regions of the brain with rich blood flow with 80% occurring in the cerebral hemispheres, 15% in the cerebellum, and 5% in the brainstem.^4^ Patients commonly present with symptoms as a result of the tumor location either by direct tumor involvement or peritumoral edema and mass effect. Clinical presenting symptoms are typically headache, focal neurologic deficit, and/or seizure. Cognitive impairment is also often seen at the time of diagnosis in up to 90% of patients with BM.^5,6^

Precision management of BM is based on the individual patient- and tumor-specific variables: tumor histology, Karnofsky performance status (KPS), prognosis, targetable mutations, number/volume of lesions and symptoms, and patient preference.

Here, we review current treatment strategies for patients with metastatic brain tumors and we highlight areas of future study.

## Pathogenesis

The main route of delivery of metastatic disease to the brain is by hematogenous spread. As a result, many metastatic lesions are located directly in the terminal “watershed regions” of arterial circulation located where tumor cells lodge in end-organ capillaries at the junction between gray and white matter.^7^ In addition, BMs of certain tumor types (prostate cancer; uterine, gastrointestinal, and breast cancers) more commonly occur in the posterior fossa.^7,8^ This phenomenon highlights the hypothesis that unique aspects of metastatic tumors, which adapt to different locations within the brain (ie, the “seed and soil” hypothesis).

Metastatic brain lesions are often molecularly and genetically distinct from the primary tumor.^9^ These tumors will typically proliferate and gain the capacity to penetrate the blood-brain barrier (BBB) and colonize the local brain microenvironment. Once through the BBB, they must then gain access to vasculature for future growth through “vascular cooption”.^10^ This allows the tumor cells to gain access to angiogenic factors, nutrients, and oxygen. In addition, BMs develop dynamic interaction with astrocytes and glial cells, which can aid in preserving BM. They may also adapt to the brain microenvironment transitioning to alternative fuel (glucose) and begin secreting factors that alter the local immune environment to shield from immune destruction.^10,11^

## Overview of Management

The management of BM has evolved over time to include many important patient-specific factors to help aid in the decision-making process. Our review includes factors from the Graded Prognostic Assessment (GPA) such as a patient’s age, KPS, disease burden and treatment response, molecular markers, and pretreatment cognitive function, in addition to size and number of metastatic lesions. Treatment options will include best supportive care with symptom management, radiosurgery, surgical resection, whole-brain radiotherapy with memantine (WBRT + M), whole-brain radiotherapy with parotid sparing, whole-brain radiotherapy with hippocampal avoidance (HA-WBRT), stereotactic radiosurgery (SRS), and systemic therapy.

## Estimation of Prognosis

A key component of BM management is an accurate estimation of prognosis, as this can guide the decision for aggressive care options versus best supportive care. The presence of BMs is usually a sign portending poor prognosis. Historically, the prognosis was estimated based on a landmark recursive partitioning analysis study that pooled patients from 3 Radiation Therapy Oncology Group (RTOG) trials utilizing several prognostic factors: age, KPS, and presence of extracranial disease.^12^ Over time, it was recognized that additional factors may play a role in improving prognostic assessment for patients and an additional GPA was derived from 4 RTOG trials.^13,14^ This analysis continues to accrue patients from multiple institutions in the United States and has since lead to the creation of the disease-specific graded prognostic assessment (DS-GPA). The DS-GPA now includes multiple factors including age, KPS, presence of extracranial disease, number of brain lesions and for lung, breast, and melanoma utilizes molecular markers. The scoring system is based on a range of 0–4.0. Although survival estimates vary by histology, approximate ranges of estimated medial survival time with 0–1 corresponding to not more than 3 months, 2–2.5 corresponding to 6 months, and 3.5–4 corresponding to more than 1 year.^15–18^ This scoring system is a discriminating diagnostic tool to guide treatment discussions with patients and can be found at brainmetgpa.com. Importantly, the ability to identify patients likely to survive more than 6 months can help to guide aggressive brain-directed therapy decisions, while patients with an estimated survival of less than 3 months may benefit from supportive care measures alone.

## Imaging

Modern management of BM requires high-quality brain imaging for both upfront therapy decision-making and assessment of response and toxicity. Our gold standard imaging modality is brain 1.5 T or 3 T magnetic resonance imaging (MRI).^19^ Additionally, efforts should be made to standardize imaging acquisition and if possible patients should be followed up with imaging performed using the same MRI imaging modality (magnet strength and protocol). This can help to minimize subjective changes based on imaging acquisition, which is most important for patients being monitored in long-term follow-up. Recommended 1.5 T MRI sequences include a 3D T1-weighted precontrast and 3D T1-weighted postcontrast series with a slice thickness of not more than 1.5 mm, a field of view of 256 mm, and 0 gap (skip) using isotropic square pixels. This will allow for high-resolution imaging that can be reliably reconstructed in the axial, coronal, and sagittal planes.

In addition, axial 2D FLAIR, axial 2D DWI, and axial 2D T2-weighted sequences should be performed using a slice thickness of not more than 4 mm, a field of view of 240 mm, and 0 gap. For 3 T MRI imaging the same sequences are recommended but with a slice thickness of 1 mm for the 3D T1 pre- and postcontrast imaging and the 2D imaging with a slice thickness of 3 mm.^19,20^

The accepted definitions of response assessment are based on response assessment criteria for BM from the Response Assessment in Neuro-Oncology Brain Metastasis (RANO-BM) working group.^19^ The working group expanded on the RECIST 1.1 criteria to help guide the assessment of metastatic brain lesions. A measurable tumor (target lesion) was considered any tumor at least 10 mm in the longest dimension. For modern MRI imaging with slice thickness as above, measurable lesions are considered at least 5 mm, care should be taken when assessing response for these smaller tumors as progression should be at least 3 mm in the longest dimension (for punctate lesions). Lesions that cannot be reliably measured and are not considered “target lesions”: bony skull metastasis, cystic only lesions, dural metastasis, and leptomeningeal disease (LMD). A partial response is considered a 30% decrease in the sum of the longest diameter of the target lesions (for at least 4 weeks), no new lesions, stable or decreasing steroid use, or stable or improving neurologic symptoms. Progressive disease is considered as at least a 20% increase in the sum of the longest diameter of a tumor (at least one lesion with an increase of 5 mm or more in the longest diameter). The stable disease includes changes that do not qualify for progression or partial response. Complete response requires resolution of all target lesions for at least 4 weeks, no new lesions, no steroid use (for neurologic symptoms), and stable to improved neurologic symptoms.^19^

LMD is characterized by the diffuse spread of metastatic disease to the meninges surrounding the brain and or spinal cord. The diagnosis is typically made with either MRI imaging demonstrating bulky dural enhancement (pachymeningeal disease),^20,21^ cranial nerve enhancement, or cerebral sulci enhancement or cerebellar folia enhancement. In addition, a cerebrospinal fluid (CSF) assessment is generally recommended for cytology or flow cytometry and neurologic examination to confirm that imaging findings correlate with neurologic deficits,^22^ although the sensitivity is low. The sensitivity of CSF sampling using a lumbar puncture for diagnosis of LMD is 50–60%, with additional sampling of up to 80%.^23^ The diagnosis and response assessment of LMD is not consistent among clinical trials and in clinical practice. The RANO working group has developed recommendations for the assessment of LMD (RANO-LM) which utilizes the neurologic findings, CSF, and imaging findings to give a score.^24^ Utilizing this scoring system is difficult and more recently the European Organization for Research and Treatment of Cancer (EORTC) Brain Tumor Group (BTG) Central Nervous System Metastasis Committee and the EORTC BTG Imaging Committee have developed a revised scorecard which can be used in clinical practice.^25^ However, it remains difficult to determine LMD response to therapy due to the difficulty in defining a radiographically measurable lesion which can be followed reliably over time.

## Radiotherapy

**Figure 1.**
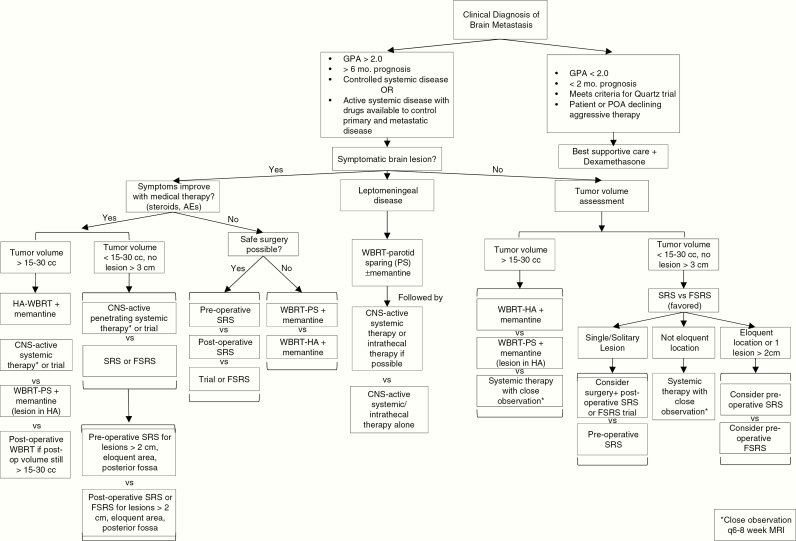
Modern precision management of patients with brain metastases based on expert consensus. *Is meant to reference the lower right box & close observation q6-8 week MRI

For patients with excellent prognosis (DS-GPA over 2), good baseline cognitive status, minimal to no neurologic symptoms, and low total intracranial disease burden, SRS is an excellent option. Many centers have adopted radiosurgery as a standard upfront option for patients due to the risk of cognitive deterioration after whole-brain radiation.^26,27^ Here, we summarize management strategies and studies that demonstrate the role of radiosurgery and whole-brain radiation. A treatment algorithm (decision tree) is proposed in Figure 1.

### Stereotactic Radiotherapy for Low-Volume Intracranial Disease

For patients with a limited number of BM, 4 or fewer lesions, the use of SRS alone is favored. This is based on 4 large phase III trials that randomized patients with limited BM to radiosurgery with or without WBRT.^26,28–30^ SRS can treat deep-seated lesions or lesions near eloquent brain structures that are not amenable to surgical resection.^31,32^ SRS is often given as a single high dose of radiation, but may also be given over 2 to 5 fractions (ie, fractionated radiosurgery) for targets that are larger-sized or near critical normal tissues such as the brainstem or the optic structures.^33,34^ Radiation dose for SRS is based on the RTOG 9005 study and is based on size with 24 Gy used for tumors less than 2 cm, 18 Gy for tumors 2.1–3 cm, and 15 Gy for tumors more than 3.0 to 4 cm.^35^ Fractionated radiosurgery (FSRS) is typically delivered with 27 Gy in 3 fractions or 25–30 Gy in 5 fractions.^33,36^ The most recent phase III trial, N0574, that randomized patients with limited BM to WBRT + SRS or SRS demonstrated there was no difference in survival. There was however a significant increase in cognitive failure for patients receiving WBRT + SRS 53% versus 20% for SRS alone at 3 months.^27^ With this evidence for patients with limited BM who have appropriate lesion size, location and prognosis should receive radiosurgery alone to spare cognitive decline while obtaining local control. Following radiosurgery, the recommended follow-up includes MRI every 2–3 months. This short interval imaging is to identify new distant intracranial tumors for salvage therapy due to the increased risk of distant failure noted with SRS alone.

### SRS for Numerous Metastasis/High-Volume Metastatic Disease

SRS and FSRS have in the past been used for limited BM, with many institutions using WBRT for patients with numerous (>4) BMs. Previous WBRT trials found that although adjunctive WBRT reduces the relative risk of intracranial disease progression by approximately 50% compared with SRS alone, it does not extend overall survival (OS) and is associated with increased risk of side effects, including neurocognitive decline.^27,29,30^ Although traditionally used to treat a limited number of tumors, prospective nonrandomized data in patients with newly diagnosed BMs suggest that up to 10 tumors with a total cumulative volume not more than 15 mL may be treated in a single session with similar efficacy and no increase in toxicity.^37,38^

**Figure 2.**
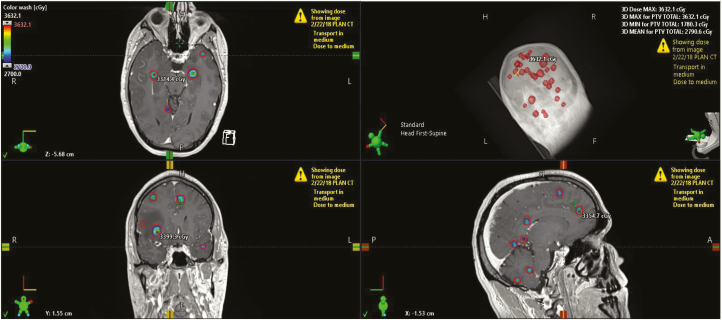
An example of a patient treated using single-isocenter multitarget radiosurgery for 30 brain lesions. The total volume of brain tumor (GTV total) is 4.7 cc. A 2–3 mm planning target volume (PTV) was used with 24 Gy prescribed to the PTV total and 27 Gy to the gross tumor volume total in 3 fractions. Treatment delivered with 5 volumetric modulated arc therapy arcs.

Recently, the National Comprehensive Cancer Network (NCCN) guidelines have recognized the utility of tumor volume plays a more important role in determining the use of SRS versus WBRT.^39^ Numerous studies support the use of tumor volume compared to the number of tumors to better predict OS (Table 1).^40–45^ The appropriate cutoff of tumor volume to determine who may benefit from WBRT rather than SRS is not well defined. Prior studies have used cutoffs ranging from 10 to 15 cc when treating multiple BMs.^37,41^ However, numerous studies have treated patients with larger volumes of tumor volume (up to 30 cc) with SRS and achieved excellent outcomes.^36,46,47^ There is also evidence that using radiosurgery results in a lower dose to the hippocampus compared to HA-WBRT.^36,48^Figure 2 demonstrates a patient with 30 brain lesions treated with radiosurgery with very little dose to the hippocampus (<4 Gy mean) and a low brain dose of 8 Gy. This may translate to improved cognitive outcomes but the prospective evaluation is needed. Importantly, patients with 5–15 BMs should be offered enrollment on CE.7, a Canadian Cancer Trials Group/Alliance/NRG clinical trial that randomizes patients to either SRS or HA-WBRT and memantine (NCT03550391).

**Table 1:** Comparison of Brain Metastasis Number of Lesions Versus Volume of Lesions in Retrospective Series

	Multivariate Analysis of Overall Survival			Citation
Patient Number	Volume of Metastasis	Number of Metastasis	Median Tumor Volume, cc	
251	*P* < .001	*P* = .2 (NS)	0.89 (0.03–22.9)	Likhacheva et al., 2013^40^
250	*P* = .003	*P* = .1 (NS)	1.2 (0.1–14.2)	Baschnagel et al., 2013^41^
201	*P* = .008	*P* = .96	4.2 (1–12.1)	Hirshman et al., 2018^42^
205	*P* = .002	*P* = .2 (NS)	6.8 (0.6–51)	Bhatnagar et al., 2006^43^
391	*P* = .0014	*P* = .19	3.41 (0.071–81.6)	Routman et al., 2018^44^
300	*P* = .004	*P* = .26	0.41 (0.01–65.01)	Emery et al., 2017^45^

In contrast to WBRT, the efficacy of SRS appears to be independent of the primary tumor type. Relatively radioresistant primary tumor histologies like renal cell carcinoma^49^ and melanoma^50^ have control rates similar to relatively radiosensitive tumor types such as breast cancer and non-small-cell lung cancer (NSCLC).^51^

Due to the excellent local control with radiosurgery in patients with numerous BMs, tumor volume appears to be a superior personalized marker for determination for survival. For patients with low overall intracranial disease burden (<15–30 cc), the use of radiosurgery may be an alternative to HA-WBRT pending further phase III data. However, the definition of low overall intracranial disease burden requires prospective validation and is not well defined. For patients with higher disease burden, the use of HA-WBRT is more appropriate given the worse OS and higher rate of distant disease/neurologic death. HA-WBRT is also more appropriate for patients with CNS-dominant disease (ie, multiple BMs with a limited volume of extracranial disease) and for patients with high BM velocity following upfront SRS.

### Radiosurgery for Small-Cell Lung Cancer

Traditionally, the standard of care for patients with small cell BM has been WBRT. These patients were excluded from our large randomized clinical trials for radiosurgery due to the high predilection for distant brain failure.^52^ However, there is early evidence that with improvements in MRI imaging and systemic therapy, particularly immunotherapy, that SRS may be appropriate for select patients.^53,54^ A recent retrospective based study of 5952 patients with small-cell lung cancer BM revealed that upfront SRS was associated with superior OS (median 10.8 vs 7.1 months, hazard ratio [HR] 0.65, 95% confidence interval [CI] 0.55–0.75, *P* < .001), which persisted on multivariate analysis controlling for comorbidities, extracranial metastases, age, race/ethnicity, and gender (HR 0.70, 95% CI 0.60–0.81, *P* < .001).^55^ Further studies are necessary to help define patient subgroups who benefit from SRS versus HA-WBRT for patients with small-cell lung cancer.

### Role of Brain Metastasis Velocity

Recently, the concept of brain metastasis velocity (BMV) was developed to identify patients who develop rapidly progressive distant brain disease. This metric has the capacity to predict the risk of developing serial distant brain relapses after salvage SRS and is strongly correlated with survival and neurologic death.^56^ BMV is defined as:

$$\text{BMW=}\frac{\text{(Total number of new brain metastases since first SRS)}}{\text{(Time (in fractions of 1 year) since first SRS)}}$$

It was observed that BMV at first or second distant brain relapse after upfront SRS predicted OS.^56^ In a larger multi-institutional validation dataset, BMV remained prognostic.^56,57^ Specifically, patients who had a BMV more than 13 BMs/year had inferior survival compared to patients with BMV less than 4 BMs/year (*P* < .0001). Interestingly, the prognostic capacity of BMV remained significant over multiple different eras of systemic therapy. BMV at first distant brain relapse was also predictive of BMV at second distant brain relapse, highlighting the ability of BMV to serve as a surrogate marker for intracranial control. The prognostic value of BMV has since been validated in several other international patient populations.^58,59^

Importantly, BMV at first or second distant brain relapse after upfront SRS predicted for neurologic death following salvage SRS.^56,57^ Neurologic death was defined as progressive neurologic decline at the time of death irrespective of the status of extracranial disease or death from intercurrent disease in patients with severe neurologic dysfunction. Patients with BMV more than 13 BMs/year were nearly 3-fold more likely to suffer neurologic death than patients with BMV less than 4 BMs/year. In addition, patients with BMV more than 13 BMs/year, neurologic death was 43% more likely than death from non-neurologic causes after salvage SRS.

These findings demonstrate the capacity of BMV following upfront SRS to distinguish a subset of patients (BMV >13 BMs/year) whose inferior intracranial control with salvage SRS significantly raises the risk of neurologic death as a primary contributor to inferior survival. Thus, the prevention of neurologic death in this high-risk population is an important treatment goal that may impact OS, especially as systemic therapies continue to improve control of the extracranial disease. BMV may allow for personalized treatment decision-making with patients who may benefit most from distant brain control. Patients with a BMV of more than 13 may preferentially benefit from WBRT versus radiosurgery for salvage therapy to prevent distant relapse. Prospective validation of this principle is needed.

### WBRT or Best Supportive Care

The importance of estimating prognosis is demonstrated in the QUARTZ trial that randomized NSCLC patients who were not eligible for surgery or radiosurgery to optimal supportive care or optimal supportive care + WBRT (20 Gy in 5 fractions). In this study, 80% of patients on each arm had a GPA of 2 or less. Importantly, the median survival for patients on this trial was quite poor at approximately 2 months for each arm.^60^ The study found that survival was not significantly different, and there was no significant reduction in quality of life with the omission of WBRT. This study demonstrates that for patients with poor performance status and uncontrolled extracranial disease for which next-line systemic therapy is not available or recommended, best supportive care in lieu of WBRT would be a reasonable consideration.^60^

### Whole-Brain Radiotherapy With Hippocampal Avoidance

A phase II trial, RTOG 0933, accrued 100 patients with BM using conformal avoidance of the hippocampus during WBRT using intensity-modulated radiotherapy for patients with BMs.^61^ This trial demonstrated a cognitive failure rate of 33% at 4 months which compared favorably relative to historical controls. This promising result led to the design of NRG CC001, a phase III trial of WBRT + M with or without hippocampal avoidance during WBRT for patients with BMs.

The NRG CC001 trial accrued 518 patients with BM to receive either WBRT + M or HA-WBRT + memantine (HA-WBRT + M). There was no difference between arms in terms of baseline cognitive function, OS (HR 1.13, 95% CI 0.89–1.44, *P* = .31), or intracranial progression (HR 1.12, 95% CI 0.90–1.39, *P* = .33).^62^ There was no difference in grade 3 or higher toxicity between the arms.

The addition of hippocampal avoidance to WBRT + M significantly prevented cognitive decline (adjusted HR = 0.74, 95% CI 0.58–0.95, *P* = .02). The difference was first seen at 4 months (62.7% HA-WBRT + M vs 54.5% WBRT + M) and maintained throughout the follow-up period and was attributable to improvements in executive function at 4 months (*P* = .01) and learning (*P* = .049) and memory (*P* = .02) at 6 months.^62^ In analyses adjusted for stratification factors, age not more than 61 years (HR 0.61, 95% CI 0.46–0.81, *P* = .0006) also predicted for prevention of cognitive failure.^62^ Test for interaction between treatment arm and age was nonsignificant (*P* = .67), suggesting that while age independently predicts for cognitive failure, the cognitive benefit of hippocampal avoidance does not differ by age.

Importantly, the addition of hippocampal avoidance to WBRT + M also preserved the patient-reported quality of life, as assessed by the MD Anderson Symptom Inventory Brain Tumor Module. Patients on the HA-WBRT + M arm experienced less symptom interference and fewer cognitive symptoms at 6 months (estimate = −1.02, *P* = .008 and estimate = −0.63, *P* = .011, respectively, Table 2).^62^ Cognitive symptom differences were driven primarily by 2 items: problems with remembering things and difficulty speaking. At 6 months, patients on the HA-WBRT + M arm had less difficulty remembering things (mean 0.16 vs 1.29, *P* = .013) and less difficulty speaking (mean −0.20 vs 0.45, *P* = .049) as compared to the WBRT + M arm. Greater improvement in fatigue at 6 months was reported in the HA-WBRT + M arm as compared to the WBRT + M arm (mean 0.93 vs −0.16, *P* = .036).^62^

**Table 2:** Cognitive Impact of Radiation Therapy

Trial	Study Arm	Patient Number	Time of Cognitive Evaluation	Cognitive Failure Rate (%)
MDACC^29^	SRS	30	4	24
	WBRT + SRS	28		52
RTOG 0614^63^	WBRT	252	3	72
	WBRT + M	256		63
RTOG 0933^61^	HA-WBRT	100	4	33
NRG CC001^62^	WBRT + M	257	4	63
	HA-WBRT	261		54
N0574^27^	SRS	111	3	20
	WBRT + SRS	102		53
N107c^26^	Surgery + SRS	66	3	21
	Surgery + WBRT + SRS	60		60

MDACC, MD Anderson Cancer Center; SRS, stereotactic radiosurgery; WBRT, whole-brain radiation; RTOG, Radiation Therapy Oncology Group; M, memantine; HA-WBRT, hippocampal avoidance-whole-brain radiation; NRG, National Surgery Adjuvant Breast and Bowel Project, Radiation Therapy Oncology Group, and Gynecologic Oncology Group.

### Whole-Brain Radiotherapy With Memantine

For patients with a large burden of intracranial disease and/or LMD, the use of WBRT is an optimal palliative treatment to improve intracranial control. Historically, the combined use of SRS and WBRT has excellent local tumor control. The main difference in these tumor-directed techniques is that SRS alone has a higher rate of distant intracranial tumor failure. In prior phase III trials, typically 32–64% of patients will have distant brain failure with the omission of WBRT.^28–30^ Therefore, patients at high risk for distant brain failure are ideal candidates for WBRT. In addition, patients with LMD have a mode of disease spread that is diffuse within the brain which makes a local treatment like SRS not useful, requiring WBRT or craniospinal irradiation for disease control. We address pachymeningeal (or dural based) disease as a separate pattern of disease that may benefit from a local treatment.^21^

When considering the use of WBRT for these situations, a standard option is to use concomitant and adjuvant memantine.^63^ A large phase III trial demonstrated that the use of memantine during WBRT and continuing for 6 months failed to meet its primary endpoint (decline in the delayed recall).^63^ However, this study was performed in an era where patient’s prognosis was poor with a median survival time of 6–8 months and 34% of patients had died before the 6 months assessment with an additional 11% of patients withdrawing consent (the primary time point for analysis).^63^ The resulting power to detect a difference may have been insufficient, the cognitive failure rate (failure on any test) at 6 months was significantly improved by approximately 11% (54% vs 65%; HR 0.78, *P* = .01).^63^ On additional analysis, it was noted that memantine significantly improved executive function (*P* = .0041), processing speed (*P* = .0137), and delayed recognition (*P* = .0149). Memantine is well tolerated with a side effect profile similar to placebo with dizziness as one of the most common side effects. In practice, a slow up-titration is started along with the initiation of radiation, beginning at 5 mg daily and increasing one a week by 5 mg with a target of reaching 10 mg of memantine twice daily.^63^ Preclinical studies suggest a benefit to starting memantine before initiating WBRT and if temporally feasible (ie, a patient seen on Friday and WBRT starts on the upcoming Monday) it is worth considering starting memantine a few days before WBRT.^64^

### WBRT With Parotid Sparing and/or Lacrimal Sparing

Historically, WBRT has been delivered with opposed lateral fields arranged to avoid the lens and encompass the entire brain using the 2D technique. This is a simple technique that can be done emergently without the need to contour any avoidance structures and relies on bony anatomy. With advances in radiation techniques and the use of CT imaging for simulation, 3D conformal therapy may be performed to improve acute toxicity in patients receiving WBRT. Several studies have prospectively evaluated various organs at risk to determine if these may show a dose-response in order to better understand toxicity related to WBRT.

In a prospective, observational cohort study 100 patients received WBRT for the treatment of BMs. Patients received 3D WBRT using opposed lateral fields covering the skull and the C1 or C2 vertebra. The proportion of patients who self-reported to be bothered quite a bit or bothered very much by xerostomia at 1 month was 50% in those with parotid V20Gy at least 47%, compared with only 4% in those with parotid V20Gy not more than 47% (*P* < .001).^65^ The xerostomia score was 23 points (95% CI 16–30, *P* < .001) at 1 month and declined over time but remained elevated with a score of 14 points (95% CI 7–21, *P* = .03) at 6 months. At 3 months, this difference was 50% versus 0% (*P* = .001). These data provide evidence with a validated xerostomia measure that keeping the parotid gland V20 not more than 47% may decrease the rate of xerostomia in patients receiving whole-brain radiation.^65^ Importantly, sparing the parotid gland may be done with modifications in the lateral fields which do not compromise the dose to the brain.

This study also included a secondary objective aiming to identify the rate of dry eye symptoms. The proportion of patients with an increase in dry eye symptoms was significantly higher at 1 month (≥1 point Subjective Evaluation of Symptom of Dryness increase) for lacrimal V20Gy at least 79% was 46% while less than 79% associated with 15% (*P* = .02).^66^ Importantly, this appears to be a previously over-looked toxicity to WBRT. However, care should be taken in attempting to spare the lacrimal glands as this toxicity measure is not validated and modifications in the field to spare lacrimal glands may compromise dose to the cribriform plate although this may be possible with HA-WBRT.

WBRT carries a relatively high risk of cognitive failure between 3 and 6 months with randomized trials demonstrating a risk of 50–70%.^26,27,29,61–63^ In Table 2 we summarize the cognitive failure rates (using different definitions of cognitive failure) for modern trials that use similar tools to measure cognitive function. This high rate of cognitive failure led to attempts to spare the hippocampus to determine if this may improve the preservation of cognitive function in patients with BM.

### Other Future Sparing Areas for Memory Protection and Imaging

**Figure 3.**
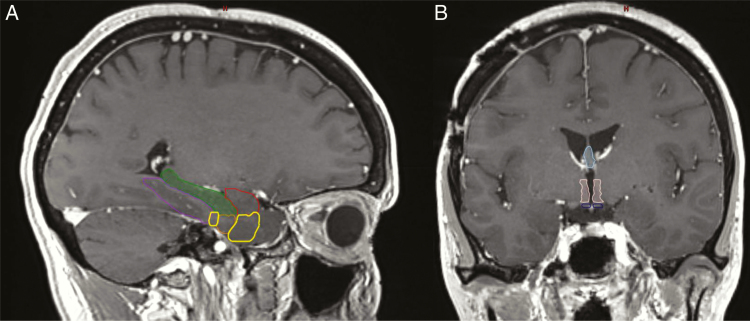
(A) Sagittal MRI demonstrating the anatomy of the medial temporal lobe structures: the amygdala (red), hippocampus (green), parahippocampal cortex (purple), perirhinal cortex (yellow), and the entorhinal cortex (orange). (B) Coronal MRI demonstrating the fornix (light blue), hypothalamus (light pink), and mammary bodies (dark blue).

Our understanding of radiation injury and its impact on memory is improving. Currently, there is level 1 data suggesting that sparing the hippocampus improves the cognitive failure rate in patients receiving whole-brain radiation. There may be other regions or pathways in the brain that are important in memory impairment and sparing these regions may further improve cognitive outcomes. There are additional subregions in the temporal lobe white matter that play an integral role in memory. The entorhinal cortex is critical for the memory, navigation, and the perception of time. The perirhinal cortex is important in the processing of sensory information for memory formation. The parahippocampal cortex is important in the recognition and coding of environmental scenes.^67^ The amygdala, fornix, and mammary bodies also impact memory as part of the limbic system. The mammary bodies are important for recollected memory and damage leads to amnesia. The amygdala is important for decision-making, emotional response, and processing memories. Both declarative and episodic memory are related to the amygdala. The fornix is a major outflow tract from the hippocampus to the diencephalon and basal forebrain and is important for recall memory.^68^ Interestingly, a large multi-institutional study found that the brainstem, bilateral thalami, hippocampi, parahippocampal gyri, amygdala, and temporal poles had a cumulative risk of harboring a BM of approximately 4–5%, suggesting that radiation to these areas could potentially be avoided with minimal risk of leaving untreated subclinical disease. Figure 3 depicts these additional important memory structures. Many are contiguous which may allow for an avoidance structure that could spare these medial temporal lobe structures and central region (hypothalamus, fornix, mammary bodies). Future studies should pursue memory sparing WBRT (MS-WBRT) addressing these other important functional regions for memory. Interestingly, a novel radiation technique known as FLASH, or ultra-high dose rate radiotherapy, is hypothesized to address this problem, it may provide adequate tumor control and spare cognitive decline. A recent study in a preclinical setting demonstrated less neuroinflammation and reduced cognitive impairment in mice.^69^

### Prophylactic Cranial Irradiation

Lung cancer has a high incidence of BMs, this predilection for brain failure led to studies attempting to prophylactically treat micrometastatic disease in the brain in patients at highest risk for BM with prophylactic cranial irradiation (PCI).^70^ The Auperin meta-analysis previously demonstrated that PCI lowered the rate of new BM by 60% and led to an OS benefit of 5% in patients who achieve remission with chemotherapy for small-cell lung cancer.^71^ Similarly, a randomized PCI trial was performed with patients with advanced-stage NSCLC which revealed no statistically significant improvement in OS, but there was a 57% decrease in the rate of new BM, and this led to a statistically significant improvement in disease-free survival of 3% at 5 years and 5% at 10 years.

A recent phase III trial for extensive-stage small-cell lung cancer failed to demonstrate a survival benefit compared to close observation with brain imaging, calling into question the indiscriminate use of PCI.^72^ Due to the risk of cognitive decline and lack of routine close interval MRI imaging in prior studies, the NCCN guidelines allow close observation with MRI imaging an alternative, especially for patients at high risk of WBRT-induced cognitive failure which includes patients with a poor cognitive baseline or patients with advanced age.^54,73,74^ A large phase III study, NRG CC003 will provide further insight into the cognitive impact of HA-PCI versus conventional PCI for patients with small-cell lung cancer, this study is actively accruing. In addition, a phase III trial (SWOG S1827) will randomize limited and extensive-stage small-cell lung cancer patients to MRI surveillance with and without PCI in an effort to evaluate whether MRI surveillance along allowing for early salvage therapy can result in similar OS and improved cognitive preservation and quality of life.

Future studies utilizing PCI may provide additional benefit as there is a clear improvement in the incidence of BM and disease-specific survival. This is most relevant for patients at high risk of BM such as small-cell lung cancer, NSCLC, and HER2+ or triple-negative breast cancer. If further improvements can be made to decrease the cognitive impact of PCI this may translate into an excellent treatment to improve survival and spare toxicity.

## Surgery

The role of surgery for patients with BM is commonly limited to large metastatic lesions at least 2cm in greatest dimension, symptomatic, or lesions that may be inducing life-threatening cerebral edema.^75^ In addition for patients with an unclear primary cancer diagnosis, surgery can obtain a histologic diagnosis or for patients with single or solitary brain disease. Historically, as many as 11% of patients with a single brain lesion may not have metastatic disease and rather have a meningioma, glioma, an infectious or inflammatory process although with more modern imaging this number is likely smaller.^76^

### Postoperative SRS and Fractionated SRS

Resection of BMs is typically performed on large lesions with mass effect causing symptoms and has been shown to have a survival benefit.^76^ However, even after gross total resection, there is approximately a 50% risk of local recurrence in the surgical bed.^77,78^ Postoperative WBRT reduces the risk of recurrence in the surgical bed by more than 50%, but these benefits have not been translated into a survival benefit, recognizing these clinical trials were not powered to assess survival.^28,77^ In an effort to avoid the acute and late toxicities of WBRT, yet improve surgical bed control, SRS to the surgical bed has been used in the postoperative setting and reported in multiple retrospective studies.^21,26,36^ Recently, a phase III trial assessed the role of postoperative SRS was that randomized 132 patients to SRS to the surgical cavity or observation after complete resection of BMs.^78^ This trial found that surgical bed control rates were significantly improved after radiosurgery compared to resection alone (12-month freedom from local recurrence 43% vs 72%; HR 0.46, *P* = .015).^78^ They also found that surgical bed control decreased as a function of increasing tumor bed size. A cooperative group, multi-institutional phase III trial, N107C/CEC.3, randomized 194 adult patients with a resected BM to either SRS or WBRT + SRS and found improved preservation of cognitive function with SRS and no difference in survival between the study arms.^26^ These phase III trials established postoperative SRS as a standard of care to improve surgical bed control (relative to observation) and represent a less toxic alternative than WBRT. Interestingly, the Alliance trial demonstrated a 60% surgical bed control following SRS, and similarly, the MD Anderson trial demonstrated poor less than 75% surgical bed control with large tumor cavities. The cause of this poor surgical bed control is likely multifactorial including radiosurgery dose (SRS dose decreases with increasing size lesions), resection cavity volume delineation, timing of radiosurgery, and surgical location/technique.

**Figure 4.**
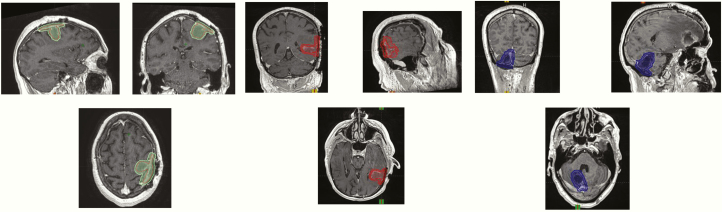
Postoperative cavity radiosurgery contouring guidelines. The complete contouring guideline may be found in the supplementary material and is also found along with the CTSU website materials for the Alliance trial A071801.

In Figure 4, we depict a sample of the contouring guideline for the A071801 trial that demonstrates the additional dural margin which is intended to lower the risk of locoregional recurrence. Currently, a larger margin is recommended along the dura to decrease the risk of marginal tumor recurrence. In addition, retrospective data support that surgical bed control is improved with FSRS compared to SRS.^34,79,80^ A clinical trial is currently underway, A071801, which randomizes patients with limited BMs with one resection bed to SRS versus FSRS. The primary endpoint is to determine if the time to surgical bed failure is increased with FSRS compared to SRS in patients with resected BM.

### Preoperative Radiotherapy

Classical leptomeningeal or pachymeningeal failure can occur after surgical resection.^20,21^ It is believed that the risk is significantly diminished with the use of adjuvant WBRT and the risk may be higher with adjuvant radiosurgery. A promising proposed strategy to decrease the risk of pachymeningeal and leptomeningeal failure followed surgical resection is preoperative radiosurgery. There are several reasons why this technique may provide benefit including improved ability to identify and contour tumor, sterilization of the tumor should any spillage occur during surgery into the cerebrospinal space, high-risk tumor locations such as the posterior fossa for iatrogenic spread, and less radiotherapy to surrounding brain as resection cavities are typically larger than the intact metastasis.^81^ Retrospective studies have demonstrated that preoperative radiosurgery has lower rates of radionecrosis and less LMD.^20,81,82^ Further prospective validation of this approach is warranted.

## Systemic Therapy for BMs

The role of systemic therapy in the management of BMs is currently evolving. Treatment strategies had previously focused on local therapeutic options such as surgery, stereotactic radiation, whole-brain radiation, or a combination. Previously the role of systemic therapy in the treatment of BMs was limited to chemotherapy which has variable CNS penetration due to the BBB. As driver mutations have been identified and targeted therapies and immunotherapies have emerged with better CNS penetration, systematic management for BMs prior to or after local treatment is now an option. We summarize here the various systemic therapies such as chemotherapy, targeted therapy, and immunotherapy that have been completed or under active investigation for a variety of solid tumors (Table 3).^83–113^

**Table 3:** Systemic Therapy Intracranial Control Rates

Population	Study Intervention	Treatment Type	*N*	Study Design	CNS Response	Reference
Breast cancer	Etirinotecan pegol	Topoisomerase inhibitor	31	Subgroup analysis of phase III	OS 4.8 months in the control group to 10 months in the group treated with etirinotecan pegol	^79^
	Tucatinib + trastuzumab + capecitabine	Small molecule inhibitor + monoclonal antibody + chemotherapy	60	Phase Ib	42%	^81^
	Tucatinib + trastuzumab	Small molecule inhibitor + monoclonal antibody		Phase 1	12%	^80^
	Lapatinib + capecitabine	Small molecule inhibitor + chemotherapy	13	Phase II	38%	^83^
	Lapatinib + capecitabine	Small molecule inhibitor + chemotherapy	30	Phase II	32%	^84^
	Lapatinib + capecitabine	Small molecule inhibitor + chemotherapy	162	Phase II	21%	^85^
	Neratinib + capecitabine	Small molecule inhibitor + chemotherapy	49	Phase II	49%	^114^
	Pyrotinib + capecitabine	Small molecule inhibitor + chemotherapy	279	Phase III	Not CNS specific	^91^
	Abemaciclib	Small molecule inhibitor	52	Phase II	25%	^92^
	Abemaciclib	Small molecule inhibitor	23	Phase II	8.7%	^115^
	Lapatinib	Small molecule inhibitor	242	Phase II	2.6–6%	^116,117^
	Neratinib	Small molecule inhibitor	40	Phase II	8%	^114^
	High-dose pertuzumab/trastuzumab	Monoclonal antibody	15	Phase II	20%	^91^
	Capecitabine + temozolomide	Chemotherapy	24	Phase I	18%	^90^
	Temozolomide + cisplatin	Chemotherapy	15	Phase II	40%	^93^
	TDM-1	Antibody drug conjugate	53	Retrospective	24.5%	^87^
	TDM-1	Antibody drug conjugate	32	Retrospective	44%	^88^
Melanoma	Dabrafenib	Serine/threonine kinase inhibitor	172	Phase II	39.2 %	^108^
	Dabrafenib + trametinib	Serine/threonine kinase inhibitor		Combi-MB	59%	^94^
	Vemurafenib	Serine/threonine kinase inhibitor			15%	^95^
	Ipilimumab	Monoclonal antibody	72	Phase II	16 %	^96^
	Ipilimumab + nivolumab	Monoclonal antibody		NCT02374242	46%	^89^
	Ipilimumab + nivolumab	Monoclonal antibody	66	Phase II	44%	^88^
	Ipilimumab + nivolumab	Monoclonal antibody	75	Phase II	56%	^98^
	Ipilimumab + nivolumab	Monoclonal antibody	19	Phase I	50%	^108^
Non-small-cell lung carcinoma	Alectinib	Small molecule inhibitor	84	Phase II	57%	^99^
	Alectinib	Small molecule inhibitor	21	Phase III (ALEX)	81%	^100^
	Brigatinib	Small molecule inhibitor	40	Phase II	78%	^115^
	Ceritinib	Small molecule inhibitor		Phase III (ASCEND-4)	NR (IC-PFS 10.7 months)	^101^
	Erlotinib	Small molecule inhibitor	48	Phase II	58.30%	^102^
	Afitinib	Small molecule inhibitor	100	Prospective compassionate use	35%	^112^
	Gefitinib	Small molecule inhibitor	41	Phase II	10%	^103^
	Lorlatinib	Small molecule inhibitor			40–75%	^104^
	Osimertinib	Small molecule inhibitor	22	Phase III (FLAURA)	91%	^113^
	Nivolumab	Monoclonal antibody	46	Phase II–III	33%	^105^
	Bevacizumab + carboplatin + paclitaxel	Monoclonal antibody + Chemotherapy	67	Phase II	61.20%	^106^
	Pemetrexed	Chemotherapy	39	Retrospective	38.40%	^107^
Melanoma/non-small-cell lung carcinoma	Pembrolizumab	Monoclonal antibody		NCT02085070	33% NSCLC, 22% melanoma	^88^
Renal cell carcinoma	Nivolumab	Monoclonal antibody	39	Phase II (Getug-AFU 26)	12%	^87^

OS, overall survival; CNS, central nervous system; NR, not reported; IC-PFS, intracranial-progression-free survival; NSCLC, non-small-cell lung cancer.

One class of systemic therapies for BMs is drugs targeting driver mutations such as tyrosine kinase inhibitors (TKIs) that penetrate the BBB with demonstrated activity in the CNS. For example, approximately 40–60% of patients with metastatic melanoma harbor a BRAF mutation, a genetic alteration that has been exquisitely sensitive to small molecule inhibitors such as vemurafenib and dabrafenib. A phase II study of dabrafenib demonstrated an intracranial response rate (RR) of 39% in previously untreated V600E BMs and 31% in progressive previously treated BMs.^115^ CNS responses have also been seen with a combination of MEK and BRAF inhibitors.^118,119^

Similarly, targeted agents also play a role in the treatment of BMs from NSCLC. About 10% of patients harbor mutations in the EGFR gene and 5% of patients have ALK translocations and CNS responses have been shown with targeted therapy.

First- and second-generation EGFR TKIs (including erlotinib, gefitinib, and afatinib) are active in the CNS with multiple retrospective studies showing RRs of more than 50% in EGFR-mutated patients.

Afatinib is an irreversible inhibitor of EGFR that has shown a CNS RR of 35% in patients who have previously been treated with erlotinib or gefitinib.^116^ Osimertinib has been showed to have a CNS overall RR was 70% with ostimertinib in the recently presented FLAURA study.^117^ Alectinib and ceritinib have both been approved for the treatment of BMs with ALK translocations. Brigatinib also demonstrates strong CNS activity.^114^

Breast cancer represents the second most frequent cause of BMs after lung cancer and is diagnosed in 10–20% of advanced cancers. Several targeted therapies have shown promise in breast cancer. There have been reports showed response of BMs with anti-endocrine therapy and there have also been recent studies looking at the role of CDK4/6 inhibitor, abemaciclib, showing activity in BMs.^91^ Therefore, it is reasonable to consider endocrine therapy prior to systemic chemotherapy. In HER2 over-expressing breast cancer, anti-HER2 agents such as trastuzumab, drug–antibody conjugate (TDM-1), and TKIs (neratinib, lapatinib) have been shown to have activity in BMs.^92–94,120,121^ In addition, the role of high-dose HER2 targeting monoclonal antibodies has shown promise using trastuzumab and pertuzumab in the PATRICIA study.^87^ Lapatinib, a TKI-targeting HER2 and EGFR, shows CNS activity. Single-agent lapatinib was found to have 2.6–6% activity^94,120^; while combination therapy with capecitabine was shown to have 21–38%.^88–90^ Neratinib is another small molecule TKI that has been found to have a RR of 8% and 49% in combination with capecitabine.^83,121^ Chemotherapy is currently the only systemic treatment option for BMs from triple-negative breast cancer, although immunotherapy trials are in development.

Another major advance in systemic therapy options for BMs is the development of immunotherapy which has shown intracranial activity. Several studies have demonstrated promising CNS activity of checkpoint inhibitors. In metastatic CNS disease from clear cell renal cell carcinoma, nivolumab had 12% RRs in the brain in a small phase II study.^122^ Pembrolizumab, another checkpoint inhibitor, had RRs of 33% and 22% in NSCLC and melanoma CNS disease, respectively.^123^ Dual administration of nivolumab with CTLA-4 antibody agent ipilimumab resulted in impressive RRs in melanoma-associated BMs with rates of complete response of 26%.^124,125^ Trials are ongoing in breast cancer to evaluate the role of checkpoint inhibitor therapy in CNS metastases including combinations with SRS (NCT03449238, NCT03807765, NCT03483012, NCT02563925).

As discussed, the role of systemic therapy with the development of small molecule inhibitors and immunotherapy is expanding. In patients with asymptomatic BMs and good performance status, starting with targeted therapy or immunotherapy is an option prior to local therapies (Figure 1). Important limitations in the existing literature regarding the use of systemic therapies for BM are the endpoints used (RRs) differ from those in most radiotherapy and neurosurgery literature (progression-free survival [PFS] and OS), the durability of response, and the use of salvage therapies like surgery and WBRT for progression are often not reported. Future studies should compare systemic therapies to current standards of surgery and radiotherapy with landmark PFS and OS endpoints. There are currently ongoing trials looking at the roles of other targeted therapies in patients who express CDK gene mutations, PI3K gene mutations, and NTRK/ROS1 inhibitors which can further expand the role of targeted therapy in BMs (NCT02896335, NCT03994796).

## Cost-effectiveness

Treatment decisions for patients with BMs are complex and individualized, requiring consideration of patient preferences, survival, risk of tumor recurrence, morbidity related to treatment and/or recurrence, as well as treatment costs. Cost-effectiveness analyses (CEA) provide a meaningful way to quantify and analyze the composite effect of these factors in order to better inform patient and stakeholder decisions.

A number of cost-effectiveness studies have shown that SRS alone is cost-effective to SRS + WBRT for patients with 1–3 BMs.^2,3^ A more recent CEA that also accounted for the increased costs of hippocampal avoidance during WBRT^126^ found that the cost-effectiveness of SRS versus HA-WBRT for patients with 1–3 metastases was highly sensitive to changes in patient life expectancy, with SRS being more cost-effective for patients with a shorter life expectancy (3–6 months) and HA-WBRT more cost-effective for subgroups with a longer life expectancy (12–24 months). Fewer studies have examined the cost-effectiveness of SRS for patients with multiple BMs. A CEA based on the results of the JLGK0901 and EORTC 229252-26001 trials found that the use of SRS versus conventional WBRT was marginally cost-effective for patients with up to 10 BMs, with an incremental cost-effectiveness ratio of $123 256 per quality-adjusted life year gained.^127^

No studies to date have examined the cost-effectiveness of the whole brain (with or without hippocampal avoidance) versus SRS alone for patients undergoing salvage treatment therapy following upfront SRS. Future studies will also be necessary to evaluate the cost-effectiveness of novel approaches for managing BMs including targeted therapies, immunotherapy, and laser interstitial thermal therapy (LITT).

## Recurrence and Progression

The response assessment for patients following tumor-directed treatment to the brain requires serial MRI and the use of the RANO criteria to define the progressive disease. In a small phase II trial, the RR for CNS disease with nivolumab was 12% for renal cell carcinoma. Should steroids fail to improve clinical symptoms or imaging findings over close interval imaging (4–6 weeks) additional options can include hyperbaric oxygen, LITT, pentoxyphiline and vitamin E, *Boswellia serrata*, and bevacizumab. Surgery plays a key role in the diagnosis of progressive disease versus treatment-related necrosis and gliosis. Surgery serves a dual role not only in diagnosis but is therapeutic with several studies demonstrating that re-operation for recurrent tumors can extend survival.^128–130^ Surgery in clinical practice tends to be the most definitive treatment for the progressive disease once other attempts to improve imaging findings or clinical symptoms have failed. With imaging or pathological evidence of disease progression, we recommend treatment based on performance status, symptoms, and tumor volume in a similar manner described in the treatment algorithm (Figure 1).

Hyperbaric oxygen increases oxygen delivery to the tissue via a hemoglobin-dependent transport mechanism and may reduce inflammation leading to improved vascularization of damaged irradiated tissue.^131^ Retrospective studies have demonstrated clinical and radiographic improvements in patients with brain radionecrosis in approximately 50% of patients.^132,133^ Pentoxifylline and vitamin E have demonstrated a clinical objective response in the edema volume.^134^

LITT delivers localized thermal energy to a zone of tissue surrounding the probe releasing localized thermal energy. This heat is then deposited through convection leading to coagulative necrosis of the lesion. Currently, there are 2 systems the Neuroblate System (Monteris Medical Inc.,) using a 12W diode and Visualase (Medtronic Inc.) using a 15W diode, both utilize MRI to localize the lesion and visualize the heat distribution.^135,136^ Currently no clinical trials have reported the utility of LITT for necrosis but there is retrospective data demonstrating symptom improvement and radiographic improvement following LITT.^136,137^

*Boswellia serrata* is a tree prevalent in India, the Middle East, and North Africa. The gummy exudate or resin obtained by peeling away the bark is commonly known as frankincense or olibanum. *Boswellia* is also referred to as Indian frankincense. There has been a placebo-controlled prospective study using this agent as an alternative to steroids in patients receiving radiotherapy. This study demonstrated a more than 75% decrease in cerebral edema (MRI response) in approximately 60% of patients.^138^ The major toxicities reported were gastrointestinal symptoms.

Bevacizumab, a monoclonal VEGF factor A antibody, was tested in a small randomized trial of patients who experienced symptomatic brain RN after radiotherapy and were randomized to either placebo or 4 cycles of bevacizumab every 3 weeks.^139^ Impressively, all patients who received bevacizumab had a radiographic response with a reduction in edema and contrast enhancement of approximately 60% in both the T1 postcontrast enhancement and the FLAIR imaging. Importantly, all patients had a corresponding reduction in neurologic symptoms/signs. In addition, no patients on the placebo arm had an initial radiographic or symptomatic response. Once patients crossed over to receive bevacizumab all patients experienced both radiographic and clinical response. Based on these data and additional institutional series, bevacizumab is considered an effective option for patients with BMs without contraindications to its use (typically risk factors for hemorrhage), who have progressive symptoms from radiation necrosis after SRS despite conservative management strategies (corticosteroids) and are not felt to be good candidates for surgical resection.^140^

## Conclusions and Future Directions

The clinical management and understanding of BM have changed substantially over time. A key improvement is the improved systemic therapy, which has led to better systemic control, longer survival, and thus an associated increased time at risk for developing BM. Crucial to the appropriate treatment of BM is a multidisciplinary team that includes neurosurgeons, medical oncologists, and radiation oncologists with greater attention paid to patient-specific factors and goals of care to determine the appropriate management. Multidisciplinary precision management of patients with BM emphasizes the maintenance of long-term survival while obtaining optimal local control to prevent neurologic symptoms and minimize the negative impact of therapy on cognition and quality of life.

## Funding

No funding was used for the published work.

*Conflict of interest statement*. The authors declare no conflicts of interest related to the published work.

## References

[1] Patchell RA The management of brain metastases. Cancer Treat Rev. 2003;29(6):533–540. 14585263 10.1016/s0305-7372(03)00105-1

[2] Hutter A, Schwetye KE, Bierhals AJ, McKinstry RC Brain neoplasms: epidemiology, diagnosis, and prospects for cost-effective imaging. Neuroimaging Clin N Am. 2003;13(2):237–250, x. 13677804 10.1016/s1052-5149(03)00016-9

[3] Posner JB Management of brain metastases. Rev Neurol (Paris). 1992;148(6–7):477–487. 1448668

[4] Arvold ND, Lee EQ, Mehta MP, et al. Updates in the management of brain metastases. Neuro Oncol. 2016;18(8):1043–1065. 27382120 10.1093/neuonc/now127 PMC4933491

[5] Clouston PD, DeAngelis LM, Posner JB The spectrum of neurological disease in patients with systemic cancer. Ann Neurol. 1992;31(3):268–273. 1637135 10.1002/ana.410310307

[6] Meyers CA, Smith JA, Bezjak A, et al. Neurocognitive function and progression in patients with brain metastases treated with whole-brain radiation and motexafin gadolinium: results of a randomized phase III trial. J Clin Oncol. 2004;22(1):157–165. 14701778 10.1200/JCO.2004.05.128

[7] Delattre JY, Krol G, Thaler HT, Posner JB Distribution of brain metastases. Arch Neurol. 1988;45(7):741–744. 3390029 10.1001/archneur.1988.00520310047016

[8] Quattrocchi CC, Errante Y, Gaudino C, et al. Spatial brain distribution of intra-axial metastatic lesions in breast and lung cancer patients. J Neurooncol. 2012;110(1):79–87. 22802020 10.1007/s11060-012-0937-x

[9] Brastianos PK, Carter SL, Santagata S, et al. Genomic characterization of brain metastases reveals branched evolution and potential therapeutic targets. Cancer Discov. 2015;5(11):1164–1177. 26410082 10.1158/2159-8290.CD-15-0369 PMC4916970

[10] Fecci PE, Champion CD, Hoj J, et al. The evolving modern management of brain metastasis. Clin Cancer Res. January 2019;25(22):6570–6580. 31213459 10.1158/1078-0432.CCR-18-1624 PMC8258430

[11] Wen PY, Loeffler JS Management of brain metastases. Oncology (Williston Park, NY). 1999;13(7):941–954, 957–961; discussion 961–962, 9. 10442342

[12] Nieder C, Mehta MP Prognostic indices for brain metastases—usefulness and challenges. Radiat Oncol. 2009;4:10. 19261187 10.1186/1748-717X-4-10 PMC2666747

[13] Sperduto PW, Kased N, Roberge D, et al. The effect of tumor subtype on the time from primary diagnosis to development of brain metastases and survival in patients with breast cancer. J Neurooncol. 2013;112(3):467–472. 23462853 10.1007/s11060-013-1083-9

[14] Sperduto PW, Shanley R, Luo X, et al. Secondary analysis of RTOG 9508, a phase 3 randomized trial of whole-brain radiation therapy versus WBRT plus stereotactic radiosurgery in patients with 1-3 brain metastases; poststratified by the graded prognostic assessment (GPA). Int J Radiat Oncol Biol Phys. 2014;90(3):526–531. 25304947 10.1016/j.ijrobp.2014.07.002 PMC4700538

[15] Sperduto PW, Yang TJ, Beal K, et al. Estimating survival in patients with lung cancer and brain metastases: an update of the graded prognostic assessment for lung cancer using molecular markers (Lung-molGPA). JAMA Oncol. 2017;3(6):827–831. 27892978 10.1001/jamaoncol.2016.3834 PMC5824323

[16] Sperduto PW, Jiang W, Brown PD, et al. Estimating survival in melanoma patients with brain metastases: an update of the graded prognostic assessment for melanoma using molecular markers (Melanoma-molGPA). Int J Radiat Oncol Biol Phys. 2017;99(4):812–816. 29063850 10.1016/j.ijrobp.2017.06.2454 PMC6925529

[17] Sperduto PW, Deegan BJ, Li J, et al. Estimating survival for renal cell carcinoma patients with brain metastases: an update of the renal graded prognostic assessment tool. Neuro Oncol. 2018;20(12):1652–1660. 30418657 10.1093/neuonc/noy099 PMC6231200

[18] Sperduto PW, Fang P, Li J, et al. Estimating survival in patients with gastrointestinal cancers and brain metastases: an update of the graded prognostic assessment for gastrointestinal cancers (GI-GPA). Clin Transl Radiat Oncol. 2019;18:39–45. 31341974 10.1016/j.ctro.2019.06.007 PMC6612649

[19] Lin NU, Lee EQ, Aoyama H, et al.; Response Assessment in Neuro-Oncology (RANO) Group Response assessment criteria for brain metastases: proposal from the RANO group. Lancet Oncol. 2015;16(6):e270–e278. 26065612 10.1016/S1470-2045(15)70057-4

[20] Prabhu RS, Turner BE, Asher AL, et al. A multi-institutional analysis of presentation and outcomes for leptomeningeal disease recurrence after surgical resection and radiosurgery for brain metastases. Neuro Oncol. 2019;21(8):1049–1059. 10.1093/neuonc/noz049 PMC6682204 30828727

[21] Cagney DN, Lamba N, Sinha S, et al. Association of neurosurgical resection with development of pachymeningeal seeding in patients with brain metastases. JAMA Oncol. 2019;5(5):703–709. 30844036 10.1001/jamaoncol.2018.7204 PMC6512273

[22] Chamberlain M, Soffietti R, Raizer J, et al. Leptomeningeal metastasis: a response assessment in neuro-oncology critical review of endpoints and response criteria of published randomized clinical trials. Neuro Oncol. 2014;16(9):1176–1185. 24867803 10.1093/neuonc/nou089 PMC4136900

[23] Nayar G, Ejikeme T, Chongsathidkiet P, et al. Leptomeningeal disease: current diagnostic and therapeutic strategies. Oncotarget. 2017;8(42):73312–73328. 29069871 10.18632/oncotarget.20272 PMC5641214

[24] Chamberlain M, Junck L, Brandsma D, et al. Leptomeningeal metastases: a RANO proposal for response criteria. Neuro Oncol. 2017;19(4):484–492. 28039364 10.1093/neuonc/now183 PMC5464328

[25] Le Rhun E, Devos P, Boulanger T, et al.; European Organisation for Research and Treatment of Cancer (EORTC) Brain Tumor Group (BTG) Central Nervous System (CNS) Metastases Committee and the EORTC BTG Imaging Committee The RANO leptomeningeal metastasis group proposal to assess response to treatment: lack of feasibility and clinical utility and a revised proposal. Neuro Oncol. 2019;21(5):648–658. 30715514 10.1093/neuonc/noz024 PMC6502503

[26] Brown PD, Ballman KV, Cerhan JH, et al. Postoperative stereotactic radiosurgery compared with whole brain radiotherapy for resected metastatic brain disease (NCCTG N107C/CEC·3): a multicentre, randomised, controlled, phase 3 trial. Lancet Oncol. 2017;18(8):1049–1060. 28687377 10.1016/S1470-2045(17)30441-2 PMC5568757

[27] Brown PD, Jaeckle K, Ballman KV, et al. Effect of radiosurgery alone vs radiosurgery with whole brain radiation therapy on cognitive function in patients with 1 to 3 brain metastases: a randomized clinical trial. JAMA. 2016;316(4):401–409. 27458945 10.1001/jama.2016.9839 PMC5313044

[28] Kocher M, Soffietti R, Abacioglu U, et al. Adjuvant whole-brain radiotherapy versus observation after radiosurgery or surgical resection of one to three cerebral metastases: results of the EORTC 22952-26001 study. J Clin Oncol. 2011;29(2):134–141. 21041710 10.1200/JCO.2010.30.1655 PMC3058272

[29] Chang EL, Wefel JS, Hess KR, et al. Neurocognition in patients with brain metastases treated with radiosurgery or radiosurgery plus whole-brain irradiation: a randomised controlled trial. Lancet Oncol. 2009;10(11):1037–1044. 19801201 10.1016/S1470-2045(09)70263-3

[30] Aoyama H, Shirato H, Tago M, et al. Stereotactic radiosurgery plus whole-brain radiation therapy vs stereotactic radiosurgery alone for treatment of brain metastases: a randomized controlled trial. JAMA. 2006;295(21):2483–2491. 16757720 10.1001/jama.295.21.2483

[31] Hussain A, Brown PD, Stafford SL, Pollock BE Stereotactic radiosurgery for brainstem metastases: survival, tumor control, and patient outcomes. Int J Radiat Oncol Biol Phys. 2007;67(2):521–524. 17097830 10.1016/j.ijrobp.2006.08.081

[32] Trifiletti DM, Lee CC, Kano H, et al. Stereotactic radiosurgery for brainstem metastases: an international cooperative study to define response and toxicity. Int J Radiat Oncol Biol Phys. 2016;96(2):280–288. 27478166 10.1016/j.ijrobp.2016.06.009 PMC5014646

[33] Minniti G, Scaringi C, Paolini S, et al. Single-fraction versus multifraction (3 × 9 gy) stereotactic radiosurgery for large (>2 cm) brain metastases: a comparative analysis of local control and risk of radiation-induced brain necrosis. Int J Radiat Oncol Biol Phys. 2016;95(4):1142–1148. 27209508 10.1016/j.ijrobp.2016.03.013

[34] Lehrer EJ, Peterson JL, Zaorsky NG, et al. Single versus multifraction stereotactic radiosurgery for large brain metastases: an international meta-analysis of 24 trials. Int J Radiat Oncol Biol Phys. 2019;103(3):618–630. 30395902 10.1016/j.ijrobp.2018.10.038

[35] Shaw E, Scott C, Souhami L, et al. Single dose radiosurgical treatment of recurrent previously irradiated primary brain tumors and brain metastases: final report of RTOG protocol 90-05. Int J Radiat Oncol Biol Phys. 2000;47(2):291–298. 10802351 10.1016/s0360-3016(99)00507-6

[36] Palmer JD, Sebastian NT, Chu J, et al. Single-Isocenter Multitarget stereotactic radiosurgery is safe and effective in the treatment of multiple brain metastases. Adv Radiat Oncol. 2020;5(1):70–76. 32051892 10.1016/j.adro.2019.08.013 PMC7004936

[37] Yamamoto M, Serizawa T, Shuto T, et al. Stereotactic radiosurgery for patients with multiple brain metastases (JLGK0901): a multi-institutional prospective observational study. Lancet Oncol. 2014;15(4):387–395. 24621620 10.1016/S1470-2045(14)70061-0

[38] Yamamoto M, Aiyama H, Koiso T, et al. Applicability and limitations of a recently-proposed prognostic grading metric, initial brain metastasis velocity, for brain metastasis patients undergoing stereotactic radiosurgery. J Neurooncol. 2019;143(3):613–621. 31140039 10.1007/s11060-019-03199-8

[39] Nabors LB, Portnow J, Ahluwalia M, et al. NCCN guidelines index table of contents discussion. 2019:143.

[40] Likhacheva A, Pinnix CC, Parikh NR, et al. Predictors of survival in contemporary practice after initial radiosurgery for brain metastases. Int J Radiat Oncol Biol Phys. 2013;85(3):656–661. 22898384 10.1016/j.ijrobp.2012.05.047

[41] Baschnagel AM, Meyer KD, Chen PY, et al. Tumor volume as a predictor of survival and local control in patients with brain metastases treated with Gamma Knife surgery. J Neurosurg. 2013;119(5):1139–1144. 23971958 10.3171/2013.7.JNS13431

[42] Hirshman BR, Wilson BR, Ali MA, et al. Cumulative intracranial tumor volume augments the prognostic value of diagnosis-specific graded prognostic assessment model for survival in patients with melanoma cerebral metastases. Neurosurgery. 2018;83(2):237–244. 28973506 10.1093/neuros/nyx380

[43] Bhatnagar AK, Flickinger JC, Kondziolka D, Lunsford LD Stereotactic radiosurgery for four or more intracranial metastases. Int J Radiat Oncol Biol Phys. 2006;64(3):898–903. 16338097 10.1016/j.ijrobp.2005.08.035

[44] Routman DM, Bian SX, Diao K, et al. The growing importance of lesion volume as a prognostic factor in patients with multiple brain metastases treated with stereotactic radiosurgery. Cancer Med. 2018;7(3):757–764. 29441722 10.1002/cam4.1352 PMC5852368

[45] Emery A, Trifiletti DM, Romano KD, Patel N, Smolkin ME, Sheehan JP More than just the number of brain metastases: evaluating the impact of brain metastasis location and relative volume on overall survival after stereotactic radiosurgery. World Neurosurg. 2017;99:111–117. 27919761 10.1016/j.wneu.2016.11.119

[46] Hughes RT, Masters AH, McTyre ER, et al. Initial SRS for patients with 5 to 15 brain metastases: results of a multi-institutional experience. Int J Radiat Oncol Biol Phys. 2019;104(5):1091–1098. 30959122 10.1016/j.ijrobp.2019.03.052

[47] Hughes RT, McTyre ER, LeCompte M, et al. Clinical outcomes of upfront stereotactic radiosurgery alone for patients with 5 to 15 brain metastases. Neurosurgery. 2019;85(2):257–263. 29982831 10.1093/neuros/nyy276

[48] Robin TP, Camidge DR, Stuhr K, et al. Excellent outcomes with radiosurgery for multiple brain metastases in ALK and EGFR driven non-small cell lung cancer. J Thorac Oncol. 2018;13(5):715–720. 29269007 10.1016/j.jtho.2017.12.006

[49] Wowra B, Siebels M, Muacevic A, Kreth FW, Mack A, Hofstetter A Repeated gamma knife surgery for multiple brain metastases from renal cell carcinoma. J Neurosurg. 2002;97(4):785–793. 12405364 10.3171/jns.2002.97.4.0785

[50] Loeffler JS, Barker FG, Chapman PH Role of radiosurgery in the management of central nervous system metastases. Cancer Chemother Pharmacol. 1999;43(Suppl):S11–S14. 10357553 10.1007/s002800051092

[51] Gerosa M, Nicolato A, Foroni R, Tomazzoli L, Bricolo A Analysis of long-term outcomes and prognostic factors in patients with non-small cell lung cancer brain metastases treated by gamma knife radiosurgery. J Neurosurg. 2005;102(Suppl):75–80. 15662785 10.3171/jns.2005.102.s_supplement.0075

[52] Aizer AA, Lee EQ Brain metastases. Neurol Clin. 2018;36(3):557–577. 30072071 10.1016/j.ncl.2018.04.010

[53] Horn L, Mansfield AS, Szczęsna A, et al.; IMpower133 Study Group First-line atezolizumab plus chemotherapy in extensive-stage small-cell lung cancer. N Engl J Med. 2018;379(23):2220–2229. 30280641 10.1056/NEJMoa1809064

[54] Rusthoven CG Small cell lung cancer: PCI uncertainty and emerging radiosurgery interest. Int J Radiat Oncol Biol Phys. 2019;103(5):1034–1035. 30900556 10.1016/j.ijrobp.2018.12.036

[55] Robin TP, Jones BL, Amini A, et al. Radiosurgery alone is associated with favorable outcomes for brain metastases from small-cell lung cancer. Lung Cancer. 2018;120:88–90. 29748022 10.1016/j.lungcan.2018.03.027

[56] Farris M, McTyre ER, Cramer CK, et al. Brain metastasis velocity: a novel prognostic metric predictive of overall survival and freedom from whole-brain radiation therapy after distant brain failure following upfront radiosurgery alone. Int J Radiat Oncol Biol Phys. 2017;98(1):131–141. 28586952 10.1016/j.ijrobp.2017.01.201

[57] Soike MH, McTyre ER, Hughes RT, et al. Initial brain metastasis velocity: does the rate at which cancers first seed the brain affect outcomes? J Neurooncol. 2018;139(2):461–467. 29740743 10.1007/s11060-018-2888-3

[58] Yamamoto M, Aiyama H, Koiso T, et al. Validity of a recently proposed prognostic grading index, brain metastasis velocity, for patients with brain metastasis undergoing multiple radiosurgical procedures. Int J Radiat Oncol Biol Phys. 2019;103(3):631–637. 30395905 10.1016/j.ijrobp.2018.10.036

[59] Fritz C, Borsky K, Stark LS, et al. Repeated courses of radiosurgery for new brain metastases to defer whole brain radiotherapy: feasibility and outcome with validation of the new prognostic metric brain metastasis velocity. Front Oncol. 2018;8:551. 30524969 10.3389/fonc.2018.00551 PMC6262082

[60] Mulvenna P, Nankivell M, Barton R, et al. Dexamethasone and supportive care with or without whole brain radiotherapy in treating patients with non-small cell lung cancer with brain metastases unsuitable for resection or stereotactic radiotherapy (QUARTZ): results from a phase 3, non-inferiority, randomised trial. Lancet. 2016;388(10055):2004–2014. 27604504 10.1016/S0140-6736(16)30825-X PMC5082599

[61] Gondi V, Pugh SL, Tome WA, et al. Preservation of memory with conformal avoidance of the hippocampal neural stem-cell compartment during whole-brain radiotherapy for brain metastases (RTOG 0933): a phase II multi-institutional trial. J Clin Oncol. 2014;32(34):3810–3816. 25349290 10.1200/JCO.2014.57.2909 PMC4239303

[62] Gondi V, Deshmukh S, Brown PD, et al. NRG oncology CC001: a phase III trial of hippocampal avoidance (HA) in addition to whole-brain radiotherapy (WBRT) plus memantine to preserve neurocognitive function (NCF) in patients with brain metastases (BM). J Clin Oncol. 2019;37(15 Suppl):2009–2009. doi:10.1200/JCO.2019.37.15_suppl.2009.

[63] Brown PD, Pugh S, Laack NN, et al.; Radiation Therapy Oncology Group (RTOG) Memantine for the prevention of cognitive dysfunction in patients receiving whole-brain radiotherapy: a randomized, double-blind, placebo-controlled trial. Neuro Oncol. 2013;15(10):1429–1437. 23956241 10.1093/neuonc/not114 PMC3779047

[64] Duman JG, Dinh J, Zhou W, et al. Memantine prevents acute radiation-induced toxicities at hippocampal excitatory synapses. Neuro Oncol. 2018;20(5):655–665. 29112734 10.1093/neuonc/nox203 PMC5892158

[65] Wang K, Pearlstein KA, Moon DH, et al. Assessment of risk of xerostomia after whole-brain radiation therapy and association with parotid dose. JAMA Oncol. 2019;5(2):221–228. 30489607 10.1001/jamaoncol.2018.4951 PMC6439567

[66] Wang K, Tobillo R, Mavroidis P, et al. Prospective assessment of patient-reported dry eye syndrome after whole brain radiation. Int J Radiat Oncol Biol Phys. 2019;105(4):765–772. 31351194 10.1016/j.ijrobp.2019.07.015 PMC7384248

[67] Squire LR, Wixted JT, Clark RE Recognition memory and the medial temporal lobe: a new perspective. Nat Rev Neurosci. 2007;8(11):872–883. 17948032 10.1038/nrn2154 PMC2323975

[68] Rolls ET Limbic systems for emotion and for memory, but no single limbic system. Cortex. 2015;62:119–157. 24439664 10.1016/j.cortex.2013.12.005

[69] Simmons DA, Lartey FM, Schüler E, et al. Reduced cognitive deficits after FLASH irradiation of whole mouse brain are associated with less hippocampal dendritic spine loss and neuroinflammation. Radiother Oncol. 2019;139:4–10. 31253467 10.1016/j.radonc.2019.06.006

[70] Gavrilovic IT, Posner JB Brain metastases: epidemiology and pathophysiology. J Neurooncol. 2005;75(1):5–14. 16215811 10.1007/s11060-004-8093-6

[71] Aupérin A, Arriagada R, Pignon JP, et al. Prophylactic cranial irradiation for patients with small-cell lung cancer in complete remission. Prophylactic cranial irradiation overview collaborative group. N Engl J Med. 1999;341(7):476–484. 10441603 10.1056/NEJM199908123410703

[72] Takahashi T, Yamanaka T, Seto T, et al. Prophylactic cranial irradiation versus observation in patients with extensive-disease small-cell lung cancer: a multicentre, randomised, open-label, phase 3 trial. Lancet Oncol. 2017;18(5):663–671. 28343976 10.1016/S1470-2045(17)30230-9

[73] Rusthoven CG, Kavanagh BD Prophylactic cranial irradiation in small-cell lung cancer. Lancet Oncol. 2017;18(7):e365. 28677568 10.1016/S1470-2045(17)30378-9

[74] Rusthoven CG, Kavanagh BD Prophylactic cranial irradiation (PCI) versus active MRI surveillance for small cell lung cancer: the case for equipoise. J Thorac Oncol. 2017;12(12):1746–1754. 28882584 10.1016/j.jtho.2017.08.016

[75] Ewend MG, Morris DE, Carey LA, Ladha AM, Brem S Guidelines for the initial management of metastatic brain tumors: role of surgery, radiosurgery, and radiation therapy. J Natl Compr Canc Netw. 2008;6(5):505–513; quiz 514. 18492462 10.6004/jnccn.2008.0038

[76] Patchell RA, Tibbs PA, Walsh JW, et al. A randomized trial of surgery in the treatment of single metastases to the brain. N Engl J Med. 1990;322(8):494–500. 2405271 10.1056/NEJM199002223220802

[77] Patchell RA, Tibbs PA, Regine WF, et al. Postoperative radiotherapy in the treatment of single metastases to the brain: a randomized trial. JAMA. 1998;280(17):1485–1489. 9809728 10.1001/jama.280.17.1485

[78] Mahajan A, Ahmed S, McAleer MF, et al. Post-operative stereotactic radiosurgery versus observation for completely resected brain metastases: a single-centre, randomised, controlled, phase 3 trial. Lancet Oncol. 2017;18(8):1040–1048. 28687375 10.1016/S1470-2045(17)30414-X PMC5560102

[79] Ahmed KA, Freilich JM, Abuodeh Y, et al. Fractionated stereotactic radiotherapy to the post-operative cavity for radioresistant and radiosensitive brain metastases. J Neurooncol. 2014;118(1):179–186. 24604750 10.1007/s11060-014-1417-2

[80] Traylor JI, Habib A, Patel R, et al. Fractionated stereotactic radiotherapy for local control of resected brain metastases. J Neurooncol. 2019;144(2):343–350. 31313060 10.1007/s11060-019-03233-9

[81] Prabhu RS, Miller KR, Asher AL, et al. Preoperative stereotactic radiosurgery before planned resection of brain metastases: updated analysis of efficacy and toxicity of a novel treatment paradigm. J Neurosurg. 2018;131(5):1–8. 10.3171/2018.7.JNS181293 30554174

[82] Prabhu RS, Patel KR, Press RH, et al. Preoperative vs postoperative radiosurgery for resected brain metastases: a review. Neurosurgery. 2019;84(1):19–29. 29771381 10.1093/neuros/nyy146

[83] Freedman RA, Gelman RS, Anders CK, et al. TBCRC 022: a phase II trial of neratinib and capecitabine for patients with human epidermal growth factor receptor 2–positive breast cancer and brain metastases. J Clin Oncol. 2019;37(13):1081–1089. 30860945 10.1200/JCO.18.01511 PMC6494354

[84] Cortés J, Rugo HS, Awada A, et al. Prolonged survival in patients with breast cancer and a history of brain metastases: results of a preplanned subgroup analysis from the randomized phase III BEACON trial. Breast Cancer Res Treat. 2017;165(2):329–341. 28612225 10.1007/s10549-017-4304-7 PMC5543189

[85] Metzger O, Barry W, Krop I, et al. Abstract P1-12-04: phase I dose-escalation trial of ONT-380 in combination with trastuzumab in patients (pts) with HER2+ breast cancer brain metastases. In: Poster Session Abstracts. American Association for Cancer Research. 2017:P1-12-04-P1-12-04.

[86] Murthy R, Borges VF, Conlin A, et al. Tucatinib with capecitabine and trastuzumab in advanced HER2-positive metastatic breast cancer with and without brain metastases: a non-randomised, open-label, phase 1b study. Lancet Oncol. 2018;19(7):880–888. 29804905 10.1016/S1470-2045(18)30256-0

[87] Lin NU, Stein A, Nicholas A, et al. Planned interim analysis of PATRICIA: an open-label, single-arm, phase II study of pertuzumab (P) with high-dose trastuzumab (H) for the treatment of central nervous system (CNS) progression post radiotherapy (RT) in patients (pts) with HER2-positive metastatic breast cancer (MBC). J Clin Oncol. 2017;35(15 Suppl):2074–2074.

[88] Lin NU, Eierman W, Greil R, et al. Randomized phase II study of lapatinib plus capecitabine or lapatinib plus topotecan for patients with HER2-positive breast cancer brain metastases. J Neurooncol. 2011;105(3):613–620. 21706359 10.1007/s11060-011-0629-y

[89] Metro G, Foglietta J, Russillo M, et al. Clinical outcome of patients with brain metastases from HER2-positive breast cancer treated with lapatinib and capecitabine. Ann Oncol. 2011;22(3):625–630. 20724575 10.1093/annonc/mdq434

[90] Sutherland S, Ashley S, Miles D, et al. Treatment of HER2-positive metastatic breast cancer with lapatinib and capecitabine in the lapatinib expanded access programme, including efficacy in brain metastases—the UK experience. Br J Cancer. 2010;102(6):995–1002. 20179708 10.1038/sj.bjc.6605586 PMC2844035

[91] Tolaney SM, Lin NU, Thornton D, et al. Abemaciclib for the treatment of brain metastases (BM) secondary to hormone receptor positive (HR+), HER2 negative breast cancer. J Clin Oncol. 2017;35(15 Suppl):1019–1019.

[92] Fabi A, Alesini D, Valle E, et al. T-DM1 and brain metastases: clinical outcome in HER2-positive metastatic breast cancer. Breast. 2018;41:137–143. 30092500 10.1016/j.breast.2018.07.004

[93] Jacot W, Pons E, Frenel JS, et al. Efficacy and safety of trastuzumab emtansine (T-DM1) in patients with HER2-positive breast cancer with brain metastases. Breast Cancer Res Treat. 2016;157(2):307–318. 27167986 10.1007/s10549-016-3828-6

[94] Lin NU, Carey LA, Liu MC, et al. Phase II trial of lapatinib for brain metastases in patients with human epidermal growth factor receptor 2-positive breast cancer. J Clin Oncol. 2008;26(12):1993–1999. 18421051 10.1200/JCO.2007.12.3588 PMC4524351

[95] Rivera E, Meyers C, Groves M, et al. Phase I study of capecitabine in combination with temozolomide in the treatment of patients with brain metastases from breast carcinoma. Cancer. 2006;107(6):1348–1354. 16909414 10.1002/cncr.22127

[96] Jiang Z, Yan M, Hu X, et al. Pyrotinib combined with capecitabine in women with HER2+ metastatic breast cancer previously treated with trastuzumab and taxanes: a randomized phase III study. J Clin Oncol. 2019;37(15 Suppl):1001–1001. 31430226 10.1200/JCO.19.00108

[97] Anders CK, Le Rhun E, Bachelot TD, et al. A phase II study of abemaciclib in patients (pts) with brain metastases (BM) secondary to HR+, HER2- metastatic breast cancer (MBC). J Clin Oncol. 2019;37(15 Suppl):1017–1017.

[98] Christodoulou C, Bafaloukos D, Linardou H, et al.; Hellenic Cooperative Oncology Group Temozolomide (TMZ) combined with cisplatin (CDDP) in patients with brain metastases from solid tumors: a Hellenic Cooperative Oncology Group (HeCOG) phase II study. J Neurooncol. 2005;71(1):61–65. 15719277 10.1007/s11060-004-9176-0

[99] Davies MA, Saiag P, Robert C, et al. Dabrafenib plus trametinib in patients with BRAFV600-mutant melanoma brain metastases (COMBI-MB): a multicentre, multicohort, open-label, phase 2 trial. Lancet Oncol. 2017;18(7):863–873. 28592387 10.1016/S1470-2045(17)30429-1 PMC5991615

[100] McArthur GA, Maio M, Arance A, et al. Vemurafenib in metastatic melanoma patients with brain metastases: an open-label, single-arm, phase 2, multicentre study. Ann Oncol. 2017;28(3):634–641. 27993793 10.1093/annonc/mdw641

[101] Margolin K, Ernstoff MS, Hamid O, et al. Ipilimumab in patients with melanoma and brain metastases: an open-label, phase 2 trial. Lancet Oncol. 2012;13(5):459–465. 22456429 10.1016/S1470-2045(12)70090-6

[102] Long GV, Atkinson V, Menzies AM, et al. A randomized phase II study of nivolumab or nivolumab combined with ipilimumab in patients (pts) with melanoma brain metastases (mets): the Anti-PD1 Brain Collaboration (ABC). J Clin Oncol. 2017;35(15 Suppl):9508–9508.

[103] Tawbi HA-H, Forsyth PAJ, Algazi AP, et al. Efficacy and safety of nivolumab (NIVO) plus ipilimumab (IPI) in patients with melanoma (MEL) metastatic to the brain: results of the phase II study CheckMate 204. J Clin Oncol. 2017;35(15 Suppl):9507–9507.

[104] Ou S-HI, Ahn JS, De Petris L, et al. Alectinib in crizotinib-refractory ALK- rearranged non–small-cell lung cancer: a phase II global study. J Clin Oncol. 2016;34(7):661–668. 26598747 10.1200/jco.2015.63.9443

[105] Peters S, Camidge DR, Shaw AT, et al. Alectinib versus crizotinib in untreated ALK-positive non–small-cell lung cancer. N Engl J Med. 2017;377(9):829–838. 28586279 10.1056/NEJMoa1704795

[106] Soria JC, Tan DSW, Chiari R, et al. First-line ceritinib versus platinum-based chemotherapy in advanced ALK-rearranged non-small-cell lung cancer (ASCEND-4): a randomised, open-label, phase 3 study. Lancet. 2017;389(10072):917–929. 28126333 10.1016/S0140-6736(17)30123-X

[107] Wu YL, Zhou C, Cheng Y, et al. Erlotinib as second-line treatment in patients with advanced non-small-cell lung cancer and asymptomatic brain metastases: a phase II study (CTONG-0803). Ann Oncol. 2013;24(4):993–999. 23129122 10.1093/annonc/mds529

[108] Ceresoli GL, Cappuzzo F, Gregorc V, Bartolini S, Crinò L, Villa E Gefitinib in patients with brain metastases from non-small-cell lung cancer: a prospective trial. Ann Oncol. 2004;15(7):1042–1047. 15205197 10.1093/annonc/mdh276

[109] Solomon BJ, Besse B, Bauer TM, et al. Lorlatinib in patients with ALK-positive non-small-cell lung cancer: results from a global phase 2 study. Lancet Oncol. 2018;19(12):1654–1667. 30413378 10.1016/S1470-2045(18)30649-1

[110] Goldman JW, Crino L, Vokes EE, et al. P2.36: Nivolumab (nivo) in patients (pts) with advanced (adv) NSCLC and central nervous system (CNS) metastases (mets). J Thorac Oncol. 2016;11(10):S238–S239.

[111] Besse B, Le Moulec S, Mazières J, et al. Bevacizumab in patients with nonsquamous non-small cell lung cancer and asymptomatic, untreated brain metastases (BRAIN): a nonrandomized, phase II study. Clin Cancer Res. 2015;21(8):1896–1903. 25614446 10.1158/1078-0432.CCR-14-2082

[112] Bearz A, Garassino I, Tiseo M, et al. Activity of pemetrexed on brain metastases from non-small cell lung cancer. Lung Cancer. 2010;68(2):264–268. 19632738 10.1016/j.lungcan.2009.06.018

[113] Haanen J, Hwu W, Martín-Algarra S Efficacy and safety of nivolumab (NIVO) alone or combined with ipilimumab (IPI) in patients with melanoma (MEL) metastatic to the brain in a phase 1 study. Presented at: Society for Melanoma Research. 2016.

[114] Camidge DR, Kim DW, Tiseo M, et al. Exploratory analysis of brigatinib activity in patients with anaplastic lymphoma kinase-positive non-small-cell lung cancer and brain metastases in two clinical trials. J Clin Oncol. 2018;36(26):2693–2701. 29768119 10.1200/JCO.2017.77.5841

[115] Long GV, Trefzer U, Davies MA, et al. Dabrafenib in patients with Val600Glu or Val600Lys BRAF-mutant melanoma metastatic to the brain (BREAK-MB): a multicentre, open-label, phase 2 trial. Lancet Oncol. 2012;13(11):1087–1095. 23051966 10.1016/S1470-2045(12)70431-X

[116] Hoffknecht P, Tufman A, Wehler T, et al. Efficacy of the irreversible ErbB family blocker afatinib in epidermal growth factor receptor (EGFR) tyrosine kinase inhibitor (TKI)–pretreated non–small-cell lung cancer patients with brain metastases or leptomeningeal disease. J Thorac Oncol. 2015;10(1):156–163. 25247337 10.1097/JTO.0000000000000380 PMC4276567

[117] Reungwetwattana T, Nakagawa K, Cho BC, et al. CNS response to osimertinib versus standard epidermal growth factor receptor tyrosine kinase inhibitors in patients with untreated EGFR-mutated advanced non–small-cell lung cancer. J Clin Oncol. 2018;36(33):3290–3297. 10.1200/JCO.2018.78.3118 30153097

[118] Planchard D, Besse B, Groen HJM, et al. Dabrafenib plus trametinib in patients with previously treated BRAF(V600E)-mutant metastatic non-small cell lung cancer: an open-label, multicentre phase 2 trial. Lancet Oncol. 2016;17(7):984–993. 27283860 10.1016/S1470-2045(16)30146-2 PMC4993103

[119] Geukes Foppen MH, Boogerd W, Blank CU, van Thienen JV, Haanen JB, Brandsma D Clinical and radiological response of BRAF inhibition and MEK inhibition in patients with brain metastases from BRAF-mutated melanoma. Melanoma Res. 2018;28(2):126–133. 29356790 10.1097/CMR.0000000000000429

[120] Lin NU, Diéras V, Paul D, et al. Multicenter phase II study of lapatinib in patients with brain metastases from HER2-positive breast cancer. Clin Cancer Res. 2009;15(4):1452–1459. 19228746 10.1158/1078-0432.CCR-08-1080

[121] Freedman RA, Gelman RS, Wefel JS, et al. Translational breast cancer research consortium (TBCRC) 022: a phase II trial of neratinib for patients with human epidermal growth factor receptor 2–positive breast cancer and brain metastases. J Clin Oncol. 2016;34(9):945–952. 26834058 10.1200/JCO.2015.63.0343 PMC5070554

[122] Flippot R, Dalban C, Laguerre B, et al. Safety and efficacy of nivolumab in brain metastases from renal cell carcinoma: results of the GETUG-AFU 26 NIVOREN multicenter phase II study. J Clin Oncol. 2019;37(23):2008–2016. 31194611 10.1200/JCO.18.02218

[123] Goldberg SB, Gettinger SN, Mahajan A, et al. Pembrolizumab for patients with melanoma or non-small-cell lung cancer and untreated brain metastases: early analysis of a non-randomised, open-label, phase 2 trial. Lancet Oncol. 2016;17(7):976–983. 27267608 10.1016/S1470-2045(16)30053-5 PMC5526047

[124] Long GV, Atkinson V, Lo S, et al. Combination nivolumab and ipilimumab or nivolumab alone in melanoma brain metastases: a multicentre randomised phase 2 study. Lancet Oncol. 2018;19(5):672–681. 29602646 10.1016/S1470-2045(18)30139-6

[125] Tawbi HA, Forsyth PA, Algazi A, et al. Combined nivolumab and ipilimumab in melanoma metastatic to the brain. N Engl J Med. 2018;379(8):722–730. 30134131 10.1056/NEJMoa1805453 PMC8011001

[126] Lester-Coll NH, Dosoretz AP, Magnuson WJ, Laurans MS, Chiang VL, Yu JB Cost-effectiveness of stereotactic radiosurgery versus whole-brain radiation therapy for up to 10 brain metastases. J Neurosurg. 2016;125(Suppl 1):18–25. 27903191 10.3171/2016.7.GKS161499

[127] Savitz ST, Chen RC, Sher DJ Cost-effectiveness analysis of neurocognitive-sparing treatments for brain metastases. Cancer. 2015;121(23):4231–4239. 26372146 10.1002/cncr.29642

[128] Sundaresan N, Sachdev VP, DiGiacinto GV, Hughes JE Reoperation for brain metastases. J Clin Oncol. 1988;6(10):1625–1629. 3171628 10.1200/JCO.1988.6.10.1625

[129] Bindal RK, Sawaya R, Leavens ME, Hess KR, Taylor SH Reoperation for recurrent metastatic brain tumors. J Neurosurg. 1995;83(4):600–604. 7674007 10.3171/jns.1995.83.4.0600

[130] Arbit E, Wroński M, Burt M, Galicich JH The treatment of patients with recurrent brain metastases. A retrospective analysis of 109 patients with nonsmall cell lung cancer. Cancer. 1995;76(5):765–773. 8625178 10.1002/1097-0142(19950901)76:5<765::aid-cncr2820760509>3.0.co;2-e

[131] Drezner N, Hardy KK, Wells E, et al. Treatment of pediatric cerebral radiation necrosis: a systematic review. J Neurooncol. 2016;130(1):141–148. 27438082 10.1007/s11060-016-2219-5

[132] Aghajan Y, Grover I, Gorsi H, Tumblin M, Crawford JR Use of hyperbaric oxygen therapy in pediatric neuro-oncology: a single institutional experience. J Neurooncol. 2019;141(1):151–158. 30426388 10.1007/s11060-018-03021-x

[133] Hart CDR, George B, Thompson CDR, Robert E The treatment of cerebral ischemia with hyperbaric oxygen (OHP). Stroke. 1971;2(3):247–250. 5165167 10.1161/01.str.2.3.247

[134] Williamson R, Kondziolka D, Kanaan H, Lunsford LD, Flickinger JC Adverse radiation effects after radiosurgery may benefit from oral vitamin E and pentoxifylline therapy: a pilot study. Stereotact Funct Neurosurg. 2008;86(6):359–366. 18854663 10.1159/000163557

[135] Sharma M, Balasubramanian S, Silva D, Barnett GH, Mohammadi AM Laser interstitial thermal therapy in the management of brain metastasis and radiation necrosis after radiosurgery: an overview. Expert Rev Neurother. 2016;16(2):223–232. 26731270 10.1586/14737175.2016.1135736

[136] Rahmathulla G, Recinos PF, Valerio JE, Chao S, Barnett GH Laser interstitial thermal therapy for focal cerebral radiation necrosis: a case report and literature review. Stereotact Funct Neurosurg. 2012;90(3):192–200. 22678505 10.1159/000338251

[137] Beechar VB, Prabhu SS, Bastos D, et al. Volumetric response of progressing post-SRS lesions treated with laser interstitial thermal therapy. J Neurooncol. 2018;137(1):57–65. 29204838 10.1007/s11060-017-2694-3 PMC5823725

[138] Kirste S, Treier M, Wehrle SJ, et al. *Boswellia serrata* acts on cerebral edema in patients irradiated for brain tumors: a prospective, randomized, placebo-controlled, double-blind pilot trial. Cancer. 2011;117(16):3788–3795. 21287538 10.1002/cncr.25945

[139] Levin VA, Bidaut L, Hou P, et al. Randomized double-blind placebo-controlled trial of bevacizumab therapy for radiation necrosis of the central nervous system. Int J Radiat Oncol Biol Phys. 2011;79(5):1487–1495. 20399573 10.1016/j.ijrobp.2009.12.061 PMC2908725

[140] Zhuang H, Shi S, Yuan Z, Chang JY Bevacizumab treatment for radiation brain necrosis: mechanism, efficacy and issues. Mol Cancer. 2019;18(1):21. 30732625 10.1186/s12943-019-0950-1 PMC6367784

